# Intratumoral microbiome and pancreatic cancer: an enabling hallmark and path to novel treatments?

**DOI:** 10.1038/s41416-025-03324-7

**Published:** 2025-12-20

**Authors:** Malte Carstensen, Lisa-Marie Philipp, Meghna Basu, Patrick Hoffmann, Jan-Niklas Klenig, Anna Maxi Wandmacher, Susanne Sebens

**Affiliations:** 1 Institute for Experimental Cancer Research, Kiel University and University Hospital Schleswig-Holstein, Campus Kiel, Kiel, Germany; 2 Section of Evolutionary Medicine, Institute for Experimental Medicine, Kiel University, University Hospital Schleswig-Holstein, Campus Kiel, Kiel, Germany; 3 Section of Evolutionary Genomics, Max Planck Institute for Evolutionary Biology, Plön, Germany

**Keywords:** Pancreatic cancer, Cancer microenvironment

## Abstract

In 2022, Hanahan integrated polymorphic microbiomes to the hallmarks of cancer, resulting in 14 overarching features that are considered fundamental to initiation and progression of cancers. It is well acknowledged that genomic instability/genetic alterations together with tumor-associated inflammation are so called “enabling hallmarks” as they drive the acquisition of the other traits. The microbiome is a key component of the inflammatory tumor stroma. Pancreatic ductal adenocarcinoma (PDAC) in particular is characterized by a pronounced stromal compartment whose role in the acquisition of malignant properties is well documented. Recent studies indicate massive alterations of the microbiome in PDAC tissues compared to healthy pancreas or precursor lesions. However, the mechanistic role of the PDAC-associated microbiome, its influence on the hallmarks of cancer, and how this relates to PDAC malignancy remain poorly understood. This raises the question of whether the tumor-associated microbiome through its direct influence on PDAC cells, their precursors, and the surrounding non-neoplastic cells promotes the acquisition of other hallmarks that drive PDAC development and progression. This perspective article outlines the current knowledge of the impact of the PDAC-associated microbiome on the hallmarks of cancer in PDAC. These current findings support the altered microbiome as a third enabling hallmark of PDAC and emphasize that further mechanistic studies are urgently needed to further substantiate its fundamental importance for this tumor entity. This knowledge will provide the basis for clinical translation to develop more effective therapeutic approaches for PDAC.

**Figure:**
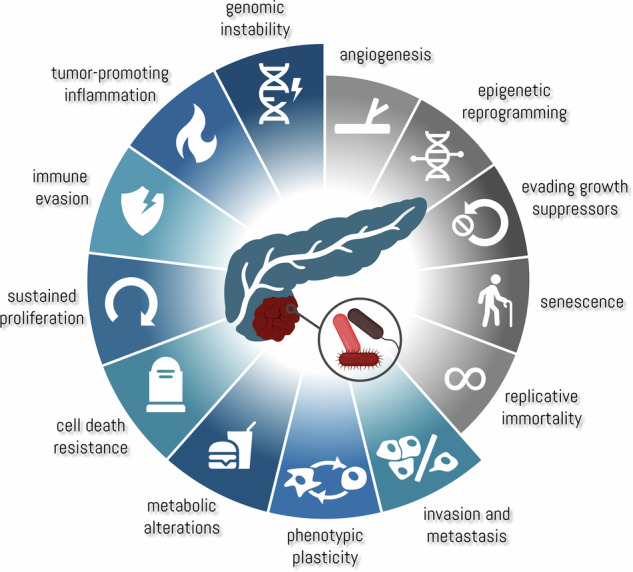
The intratumoral microbiome in PDAC exhibits numerous interactions with the hallmarks of cancer. Hallmarks indicated in blue have demonstrated interactions with the microbiome, while others still remain underexplored. These extensive interactions substantiate the role of the intratumoral microbiome in PDAC as an enabling hallmark, underlining its potential as a therapeutic target. Partially created with *biorender.com*.

The intratumoral microbiome in PDAC exhibits numerous interactions with the hallmarks of cancer. Hallmarks indicated in blue have demonstrated interactions with the microbiome, while others still remain underexplored. These extensive interactions substantiate the role of the intratumoral microbiome in PDAC as an enabling hallmark, underlining its potential as a therapeutic target. Partially created with *biorender.com*.

## Introduction

Pancreatic ductal adenocarcinoma (PDAC) is still one of the most fatal cancers due to its aggressive nature and high therapy resistance. Besides characteristic genetic alterations (e.g., in *KRAS*), PDAC is marked by a pronounced tumor microenvironment (TME) composed of inflammatory and immune cells as well as the microbiome. The latter is altered in PDAC compared to the physiological pancreas, and is associated with different outcomes, depending on its composition [1]. Given these associations, the microbiome may be regarded as an enabling hallmark of PDAC [2]. This perspective article briefly outlines the current knowledge on how the tumor-associated microbiome impacts the other hallmarks of cancer in PDAC, referring to excellent reviews and some original articles. Finally, we provide a perspective on the further steps required to emphasize this role, providing the basis for improved therapeutic concepts. When mentioned in the text, the hallmarks of cancer are highlighted in bold.

## The intratumoral microbiomes’ impact on hallmarks of cancer in PDAC

The induction of **genomic instability**, in conjunction with mutations, constitutes one carcinogenic mechanism of the intratumoral microbiome. Some bacteria synthesize substances capable of inducing DNA damage, like *Escherichia coli* (*E. coli*) colibactin [3]. The release of these substances can compromise the integrity of host DNA, which has been demonstrated to increase the risk of oncogenic changes in the respective colonized tissue [4]. For instance, the abundance of anaerobic *Fusobacterium nucleatum* (*F. nucleatum*), present in the oral cavity under physiological conditions, is highly increased in PDAC tissues, where it can induce DNA double strand breaks and increase the mutational burden [1, 5]. Furthermore, in other cancers like colorectal cancer (CRC), infection with *F. nucleatum* results in impairment of DNA repair mechanisms, leading to additional destabilization of DNA and increased mutation rates [6]. In a CRC mouse model, the bacterium promotes E-cadherin/β-catenin activation to upregulate checkpoint kinase 2, which induced additional DNA damage [7]. Since the various effects of *F. nucleatum* on PDAC are similar to its effects on CRC, it is reasonable to assume that the bacterium influences the genomic stability in PDAC as well [5, 8–12]. Studies investigating specifically this hallmark in PDAC in response to *F. nucleatum* infection have to be performed to confirm the assumption.

In addition to their impact on genomic instability, intratumoral bacteria can influence **sustained proliferative signaling** in tumor and stroma cells, as well as **inflammation** through induction of cytokine secretion [8]. *F. nucleatum* infection leads to increased IL-8, GM-CSF, and MIP-3a secretion, as well as stimulation of the JAK/STAT and MAP kinase pathways, resulting in increased proliferation and migration [13]. Furthermore, mutations caused by *F. nucleatum* stimulate the Ku70/p53 signaling pathway, and consequently the proliferative activity of infected tumor cells [5]. Besides pro-proliferative effects of *F. nucleatum*, bacterial infection with e.g., *Helicobacter pylori* (*H. pylori*) can lead to increased levels of IL-8 and VEGF, as well as an increase of the transcription factor NF-κB, further impacting cell proliferation and inflammation [14]. Similarly, *Porphyromonas gingivalis* (*P. gingivalis*), enriched in PDAC tissues compared to physiological controls, contributes to inflammation through activity of gingipain proteases [15]. Bacteria-induced inflammation is not limited to particular species; rather, it is induced by most bacteria bearing pathogen-associated molecular patterns. For instance, lipopolysaccharide (LPS) activates the PI3K-Akt-mTOR pathway in PDAC cells and activates the inflammasome [16, 17]. In *KRAS*-mutant mice, LPS stimulation promotes NF-κB-mediated development of pancreatic intraepithelial neoplasia, precursor lesions of PDAC [18]. Another study demonstrated that sterile bile juices inhibited PDAC cell growth. However, addition of bacteria to the bile juice restored cell growth, while bacterial conditioning did not [19]. In addition to these cancer promoting effects, some bacteria can have beneficial effects for patients. *Pseudomonas aeruginosa* produces mannose-sensitive hemagglutinin lectin, which leads to cell cycle arrest in PDAC, but not normal pancreatic epithelial cells [20]. Overall, these findings indicate that microbes can impact tumor cell growth and inflammation in a direct and paracrine fashion, as well as in a promoting and inhibiting manner, in dependence on the species.

The TME, as well as the tumor cells, also impact phenotype and activity of immune cells. Cancer cells evolve mechanisms to **evade destruction by immune cells** and promote an immunosuppressive and pro-tumorigenic environment, a process mediated by the intratumoral microbiome [1]. Returning to *F. nucleatum* and *P. gingivalis*, infection of PDAC cells in vitro or in vivo results in bacterial invasion to the gut and tumor, inducing a pro-inflammatory TME enriched with neutrophils, advancing further progression by chemokine release [8, 21]. Moreover, microbial diversity within PDAC tissues correlates with immune cell profiles. Tumors with more diverse microbiomes are associated with greater infiltration of activated cytotoxic T lymphocytes (CTL) and improved survival after tumor resection. Accordingly, fecal microbiome transfers (FMT) from human long-term survivors in PDAC mouse models enhance CTL invasion and pro-inflammatory cytokine expression, while microbiomes from short-term survivors promote regulatory T helper cells and myeloid-derived suppressor cells [22]. Gut microbiome depletion in PDAC-bearing mice increases effector T cell infiltration and IFNγ production, while the number of IL-17a+ and IL-10 + T cells decreases, underscoring the immunosuppressive function of the microbiome. Ablation also led to polarization of macrophages to an M1-like phenotype, resulting in slower tumor growth, driven primarily by T cells [1, 22].

Cancer cells not only evolve strategies to evade immune cells, but also to **resist cell death** [2]. It was shown that intracellular *P. gingivalis* protects PDAC cells from reactive oxygen species-induced cell death under nutrient stress conditions [21]. Additionally, this hallmark represents a key mechanism of therapy resistance [2]. Gemcitabine is a cornerstone chemotherapeutic agent in PDAC treatment, inducing cell death. However, the efficacy of Gemcitabine in PDAC patients is often limited due to the tumor’s intrinsic and acquired resistance mechanisms, potentially influenced by the intratumoral microbiome [23]. Geller et al. demonstrated that *Gammaproteobacteria* induce Gemcitabine resistance, converting Gemcitabine into its inactive form by expression of a long isoform of cytidine deaminase [24]. This resistance was reversed by the antibiotic Ciprofloxacin, underlining the influence of the bacteria. Even though these findings were obtained in a colon cancer mouse model, the study also revealed that 76% of analyzed PDAC tissues contained predominantly *Gammaproteobacteria*, supporting a role of these intratumoral bacteria in PDAC drug resistance. Indeed, antibiotic treatment prior to gemcitabine therapy, but not other cytostatic drugs, led to improved survival outcomes for PDAC patients [25]. This connection was further expanded to the evolution of therapy resistance, when *E. coli* rapidly adapted to gemcitabine treatment in a PDAC spheroid coculture model [26].

Besides the pronounced TME, PDAC is characterized by a high tumor cell **plasticity** contributing to tumor heterogeneity [27]. Tumor cell plasticity is mediated by Epithelial-to-Mesenchymal-Transition (EMT), a key mechanism to confer a motile and invasive phenotype, thereby promoting **metastasis**. Cancer stem cells (CSCs) further contribute to tumor cell plasticity and are regarded as essential for tumor initiation and progression [28]. It seems plausible that cancer cell plasticity in PDAC is influenced not only by CSCs and EMT, but also by additional factors such as stromal and immune cells and the distinct composition of the tumor microbiome. In different cancer entities, e.g., colorectal, bladder or breast cancer, a crucial association between microorganisms, EMT and CSCs was demonstrated [29]. It was reported that *H. pylori* infection induces EMT-like changes and high expression of the gastric CSC marker CD44 in vitro, leading to increased cell migration, invasion and tumor sphere formation [30]. Many PDAC tissues show presence of *H. pylori*, however the bacterium has not been clearly associated with patient outcome in this cancer entity [31]. Further knowledge about the mechanistic influence of the microbiome on PDAC progression is limited. Nevertheless, it was already shown that a tumor-associated microbiome can induce signaling pathways that cause activation of different EMT transcription factors and thus promote plasticity in PDAC [32]. It is reasonable to assume that the microbiome also influences CSCs, further impacting cancer cell plasticity. Regarding metastasis, Jeong et al. examined the microbiome detectable in extracellular vesicles derived from PDAC and corresponding non-cancerous pancreatic tissues [33]. They identified *Tepidimonas* enriched in tumor tissue and describe a positive correlation of lymph node metastasis with increased prevalence of *Comamonas* and *Turicibacter* in the tumor tissue. Treatment of PDAC cell lines with *Tepidimonas*-conditioned medium increased migration and upregulated expression of the EMT transcription factors *SNAIL* and *TWIST*. In another study, several bacterial species were enriched in the bile of patients with lymph node metastasis compared to non-metastasized PDAC [34]. Still, little mechanistic data exists elucidating the pathophysiological effects of intratumoral infection with individual bacterial species on invasion and potential metastasis capacities of PDAC cells. Udayasuryan et al. infected PDAC cells and non-malignant pancreatic epithelial cells with *F. nucleatum*, leading to elevated levels of cytokines in both cancerous and non-cancerous cells [8]. Interestingly, autocrine and paracrine exposure to these cytokines resulted in increased migration and invasion of malignant but not non-malignant pancreatic epithelial cells. Some aspects of this study were independently confirmed, as infection with *F. nucleatum*, but not with *P. gingivalis*, increased CXCL1 and IL-8 secretion by PDAC cell lines, resulting in increased migration and invasion in vitro [10]. In patient-derived tumor samples, CXCL1 expression was significantly higher in the *F. nucleatum* infected group, underlining a potential relevance of the in vitro findings for the clinical context. In a mouse model of peritoneal metastasis, bile juice pre-incubated with live *Enterococcus fecalis* (*E. faecalis*) or *Streptococcus oralis* markedly enhanced the growth of peritoneal metastasis, underscoring the promoting effect of certain bacteria on malignant phenotypes in PDAC [19].

**Deregulation of cellular metabolism** is another hallmark of cancer observed also in other pathological conditions such as obesity, metabolic syndrome and diabetes, all being associated with the incidence of PDAC [35]. The mechanism that connects these diseases involve promotion of chronic inflammation, the influence of adipose tissue-derived soluble mediators on *KRAS* signaling, and the disruption of metabolic pathways [36, 37]. These interlinked metabolic pathways, that may be initiated by one of the conditions, might lead to a higher susceptibility towards PDAC development. Of note, an altered microbiome has been implicated in the above-mentioned metabolic diseases [38]. High-fat diets, which are characteristically associated with metabolic dysregulation, can lead to a compromised intestinal barrier, resulting in leakage of bile acids into the circulatory system and initiating systemic inflammation and tumorigenesis [39]. Other bacterial metabolites affected by both metabolic dysregulation and PDAC tumorigenesis are short-chain fatty acids (SCFAs). SCFAs can reduce cancer incidence by inhibiting cell growth and migration and inducing apoptosis. In PDAC, probiotics and butyrate producing bacteria are reduced [39]. Infection with *H. pylori*, enriched in PDAC tissues, also result in metabolic dysregulation, as the bacteria produce nitrosamines and other carcinogens that can potentially induce genomic instability [40]. One of the key repercussions of metabolic dysregulation, compromised intestinal permeability, can lead to direct interlinking of the gut microbiome to the PDAC associated microbiome. The translocation of opportunistic pathobionts like *E. faecalis* from the gut to PDAC tissues, can ensure a continuous channel of colonization, resulting in continued influence on the tumorigenic conditions in PDAC [41]. *E. faecalis* is known to polarize colon macrophages into a pro-inflammatory phenotype, leading to endogenous genomic instability and cellular transformation in IL10-/- mice [42]. Interestingly, Pushalkar et al. observed bacterial migration of *E. faecalis* and *E. coli* to the pancreas upon oral administration in wild type mice, pointing towards the possibility of a similar mechanism in PDAC [43].

## Future directions

This perspective article highlights the extensive and intricate web of interactions between the tumor-associated microbiome and other hallmarks of cancer in PDAC (Table 1). As integral part of the TME, the intratumoral microbiome induces **genetic instability**, impacts **proliferative signaling** and confers **resistance to cell death**. Through mechanisms of **inflammation**, it further shapes the TME to support cancer cell survival and progression, while impairing immune function and enabling **immune evasion**. In addition, bacterial interactions and metabolites have been shown to alter the tumor cell **metabolism** with implications for PDAC progression. Cancer cell motility is impacted by intratumoral bacteria, promoting **metastasis**. There is growing evidence that the microbiome also promotes cancer cell **plasticity**, thereby contributing to tumor heterogeneity and aggressiveness of PDAC, although further research is needed to translate key findings from other cancer entities to PDAC.

**Table 1:** Multiple bacterial taxa have been investigated for their impact on PDAC progression.

Taxon	Clinical association	Affected hallmark	Mechanistic impact	Refer-ences
Fusobacterium nucleatum	Associated with PDAC and short survival	**↑** Tumor-promoting inflammation **↑** Immune evasion **↑** Sustained proliferation **↑** Metastasis	Secretion of cytokines (GM-CSF, CXCL1, IL-8, MIP-3α) boost PDAC cell proliferation, migration and invasiveness	[6]
		**↑** Sustained proliferation	Stimulation of the Ku70/p53 pathway, increasing proliferation	[5]
		**↑** Immune evasion	Secretion of CXCL1, resulting in recruitment of immunosuppressive myeloid-derived suppressor cells and reduction of CD8 + T cell infiltration	[10]
		**↑** Genomic instability	Induction of double strand breaks and upregulation of checkpoint kinase 2	[5, 7]
Porphyromonas gingivalis (P. gingivalis)	Associated with PDAC and precursor lesions	**↑** Phenotypic plasticity **↑** Cell death resistance	Increased expression of SOX9, CK19; protection against ROS	[21]
		**↑** Sustained proliferation	Development of PanIN through increase of proliferation marker expression	[48]
		**↑** Tumor-promoting inflammation	Inflammation by expression of gingipain proteases	[15]
bacteria expressing cytidine deaminase (particularily Gammaproteo-bacteria, Aggregatibacter actinomycetem-comitans, P. gingivalis, Streptococcus mutans)	Associated with short survival after chemotherapy	**↑** Cell death resistance	Both long- and short-form cytidine deaminase metabolize gemcitabine, reducing treatment efficacy	[24]
gram negative bacteria expressing LPS	Associated with short survival	**↑** Tumor-promoting inflammation	Enhance PDAC viability through induction of the inflammasome in macrophages and PDAC cells, increasing the expression of IL-1ß and TNFα	[17]
diverse microbiome (also modulated by fecal matter transplantation)	Associated with long term survival	**↓** Immune evasion	Modulates tumor growth and immune infiltration	[22]
lactic acid bacteria (particularily Lactobacillus species)	Associated with long term survival	**↓** Immune evasion	Reversal of P. gingivalis induced proliferation and inflammation;decrease of Gal-3 and PD-L1 expression on cancer cells	[48]
Escherichia coli (pks+)	No clear association	**↑** Genomic instability	Express colibactin, which can induce DNA damage	[3]
Helicobacter pylori	No clear association	**↑** Tumor-promoting inflammation **↑** Sustained proliferation	Increases levels of IL-8, VEGF	[14]
Pseudomonas aeruginosa	No clear association	**↓** Sustained proliferation **↓** Cell death resistance	Produces a mannose-sensitive hemagglutinin lectin, which induces cell cycle arrest and apoptosis through inhibition of the EGFR/Akt/ERK signaling pathway	[20]

Associations in the clinical context, interactions with other hallmarks of cancer, the underlying mechanisms and the respective original articles are listed in the table. PDAC, pancreatic ductal adenocarcinoma; PanIN, pancreatic intraepithelial neoplasia.

While the influence of the microbiome on these distinct hallmarks is established in PDAC, the impact on the other hallmarks remains underexplored. Cellular **senescence** can promote the progression of cancer through a senescence-associated secretory phenotype. Having demonstrated the influence of the microbiome on PDAC cell growth, it can be speculated that certain bacteria have a growth-inhibiting impact by which malignant cells acquire a senescent/quiescent phenotype. The association between the intratumoral microbiome and **angiogenesis** in PDAC remains to be elucidated. However, secretion of cytokines like IL-8 in response to bacterial infection may link these hallmarks. Connections to **epigenetic reprofiling,** **replicative immortality**, and **evasion of growth suppressors** are not yet well understood.

In summary, the rapidly expanding body of evidence strongly supports the view that the polymorphic microbiome has to be regarded as an enabling hallmark of cancer in PDAC. To further substantiate the enabling role of the microbiome in this disease, a deeper understanding of the underlying mechanistic interactions and the identification of beneficial and detrimental microbial communities are mandatory to transfer this knowledge into effective therapeutic concepts that could improve prognosis of PDAC patients.

To date, no microbiome-targeted therapy has been implemented in the clinical management of PDAC. However, data from preclinical models and retrospective analyses indicates its potential for future improvements in the treatment of PDAC patients.

A straightforward approach involves antibiotic therapy, ablating large portions of the microbiome across multiple organs. Retrospective analyses revealed an increase of overall survival in PDAC patients receiving gemcitabine, but not other cytostatic drug treatment [25]. In a mouse model, antibiotic-mediated microbiome ablation enhanced T cell infiltration to the TME and resolved immune checkpoint blockades [43, 44]. However, broad ablation comes with adverse side effects for the patients’ overall health. Furthermore, the resulting reduction in intratumoral alpha diversity has been associated with worse patient outcomes, and the loss of beneficial regulatory bacteria allows for uncontrolled recolonization [22]. This challenge could be addressed through targeted recolonization after ablation. Riquelme et al. demonstrated that orthotopic PDAC-bearing mice significantly benefited from FMT from human long-term survivors [22]. Indeed, two ongoing phase I clinical trials currently investigate the effect of FMT on survival of patients with either resectable (ID: NCT04975217) or non-resectable (ID: NCT06393400) PDAC [45, 46].

Additionally, studies have investigated the therapeutic potential of specific probiotic bacteria, particularly lactic acid bacteria. Oral administration of *Lactobacillus* or *Megasphaera* reduced progression of xenograft and orthotopic PDAC or pancreatic tumors in mice, respectively [47, 48]. However, use of singe-species probiotics can reduce microbial diversity, induce dysbiosis, and pose a risk of infection in immunocompromised patients [49]. Further research is required to develop complex formulation of probiotic bacteria before progressing to clinical trials.

Beyond antibiotics, bacteriophages offer potential to specifically target oncobacteria in PDAC. In a recent study, the *F. nucleatum* targeting bacteriophage ØTCUFN3 inhibited tumor cell growth in vitro and reduced progression in xenograft models of CRC [50]. Although no such studies have been performed in PDAC yet, this strategy seems to be also applicable to PDAC based on the pro-tumoigenic role of *F. nucleatum* in this tumor entity [5, 8, 10].

As a final note, the rapid adaptability of the microbiome can drive resistance to therapy regimens, as demonstrated in the context of gemcitabine resistance [26]. Therefore, data on the effects of treatments on the tumor microbiome are needed. In cases where patients relapse after primary tumor resection and subsequent treatment, and resection of the recurrent tumor becomes clinically feasible in the future, the microbiome of both specimens should be analyzed. In addition, in animal models, the compositional changes of the intratumor microbiome should be investigated and considered.

The therapeutic potential of microbiome-targeting strategies in PDAC is considerable. Their clinical implementation requires comprehensive research that further unravels the interactions between the tumor microbiome and the other hallmarks of cancer and how these impact PDAC progression and therapy responses.
